# Minimally Invasive Ablative Therapies for Definitive Treatment of Localized Prostate Cancer in the Primary Setting

**DOI:** 10.1155/2011/394182

**Published:** 2010-12-05

**Authors:** Eugene W. Lee, William C. Huang

**Affiliations:** Department of Urology, New York University School of Medicine, NY 10016, USA

## Abstract

Traditionally, the patient with a new diagnosis of localized prostate cancer faces either radical therapy, in the form of surgery or radiation, or active surveillance. A growing subset of these men may not be willing to accept the psychological burden of active surveillance nor the side effects of extirpative or radiation therapy. Local ablative therapies including cryotherapy, high-intensity focused ultrasound, and vascular-targeted photodynamic therapy have emerged as a means for minimally invasive definitive treatment. These treatments are well tolerated with decreased morbidity in association with improvements in technology; however, long-term oncologic efficacy remains to be determined.

## 1. Introduction

According to the recently updated 2007 AUA Guideline for the Management of Clinically Localized Prostate Cancer, the standard management for localized prostate cancer includes radical therapy such as, prostatectomy or radiation therapy, or active surveillance [1]. Routine use of PSA screening has increased the number of relatively young men with low-stage, low-risk disease, who may not be willing to accept the potential side effects of traditional definitive treatment [2]. Although active surveillance is an option, these men may be unwilling to accept the psychological burden associated with this approach. Furthermore, a recent study of postprostatectomy patients meeting criteria for active surveillance showed that a significant number of these patients had higher-risk disease on final pathology with 17% BCR at 5 years (defined as 3 consecutive PSA rises with peak >0.15 ng/dL) [3]. As a result, the conventional choice between radical therapy and active surveillance is not palatable for a growing subset of men diagnosed with clinically localized prostate cancer, and this has led to a greater interest in less invasive therapies that may provide cancer control with potentially decreased morbidity. Minimally invasive ablative therapies employ a variety of energy sources to ablate prostate tissue in the outpatient setting, using advanced imaging technology and protective devices to reduce treatment-related morbidity. Cryotherapy, high-intensity focused ultrasound (HIFU), and vascular-targeted photodynamic therapy (VTP) have all been used in this setting as primary therapy with varying degrees of experience.

## 2. Cryotherapy

Cryotherapy destroys prostate tissue by freezing cells down to extremely low temperatures (−40°C). Cryoprobes placed under TRUS guidance mediate extraction of heat from the prostate resulting in cell death via formation of intracellular ice as well as by induction of the apoptosis and inflammatory cascade [4]. A recent AUA Best Practice Statement describes cryotherapy as a reasonable therapeutic option in both the primary and salvage settings [5], and with a projected 15,000 procedures expected to be performed in USA in 2010 [6], it is the most widely used ablative therapy with the largest body of published data.

Cryotherapy is not a new tool for urologic oncologists—the first cryosurgical probe system using liquid nitrogen was described in 1961 by Cooper and Lee [7] and was initially used to treat benign prostatic hypertrophy by Gonder et al. [8]. In 1972, Flocks reported using a perineal incision to perform cryosurgical ablation of prostate cancer [9]. However, due to crude temperature-monitoring measures for ice ball control (digital rectal guidance or direct vision), there were high rates of rectourethral fistulas, urinary incontinence, and strictures. With the introduction of TRUS guidance by Onik [10], the modern era of cryotherapy for prostate cancer was initiated. Continued technological advancements over subsequent platforms, including thermocouple devices to monitor temperature, urethral warming to prevent fistula [11], argon gas facilitating the use of small cryoprobes passable through a brachytherapy grid [12], and the concurrent development of more sophisticated imaging and computer software for optimal probe placement, have all contributed to minimizing the morbidity of the procedure.

## 3. Selection Criteria

The recently published AUA Best Practice Statement on Cryosurgery [5] states that cryotherapy is appropriate for men with clinically organ-confined disease of any grade with a negative metastatic evaluation. Cryotherapy may be best suited for men who do not wish to undergo radical prostatectomy or radiation therapy due to side effects or because of comorbidities or conditions which make them poor candidates for either conventional surgery or radiation. Although clinical T3 disease has been treated in many reports, there are limited outcomes data for this, and therefore the role of cryotherapy in this setting remains undetermined. Best results are seen with a PSA < 10 ng/mL, and patients with high-risk disease may require multimodal therapy. Prostate gland volume can be a limiting factor, as the gland must be encompassed within the ice ball. Therefore, most reports limit prostate size to 40–60 cc. Although it has not been shown to improve cancer control, neoadjuvant androgen deprivation therapy has been used to shrink the gland to facilitate cryotherapy when a larger gland is present. A history of TURP is a relative contraindication as these patients are at increased risk of urethral sloughing.

## 4. Definition of Treatment Success

Cryotherapy has the largest body of literature of the minimally invasive ablative therapies. However, there is a lack of consensus on the definition of biochemical failure for cryotherapy, and this is the greatest limitation in defining treatment success and drawing comparisons to other treatment modalities. Published reports thus far have used PSA cut-offs from 0.1, 0.4, 0.5, and 1.0 ng/mL as well as the old ASTRO definition of 3 consecutive PSA rises, with most recent studies adopting the new ASTRO Phoenix criteria of nadir plus 2 ng/mL [13–18]. However, the Phoenix criteria authors specifically stated that it was not to be used for cryotherapy [19]. On the other hand, although cryotherapy technically ablates prostatic tissue, the standard for radical prostatectomy may not be appropriate because urethral warming technology invariably preserves periurethral prostatic tissue [20]. The appropriate cutoff level to guide treatment remains to be determined. The Cryo On-Line Data Registry (COLD registry) is a multicenter database pooling data from both academic and community centers, and the maturation of this large data set will hopefully provide answers [21].

While we currently lack such a cutoff value, it has been demonstrated that the lower the PSA after cryosurgery, the greater the likelihood that PSA will remain stable and posttreatment biopsies negative (although the value of posttreatment biopsy is questionable due to the effect of sampling error) [13, 22, 23]. In 2009, Levy applied the Phoenix criteria to 2,427 men from the COLD registry to demonstrate that a PSA nadir of 0.5 or less resulted in a favorable biochemical disease-free survival (bDFS), especially for low-risk disease [14]. 

## 5. Outcomes

Several series have greater than 5-year followup, reporting bDFS rates using various definitions for success and with results stratified according to D'Amico risk groupings. The 5-year bDFS rates range from 65% to 92% for low risk, 69% to 89% for intermediate risk, and 45% to 89% for high-risk disease [15–17, 21, 24]. The largest series is reported by Jones et al. using 1,198 patients from the COLD registry. At 5 years, using Phoenix criteria, the bDFS was 91, 79, and 62 percent, respectively [21]. The series with longest followup is reported by Cohen, who evaluated 204 patients with a median followup of 12.55 years. Using Phoenix criteria, bDFS was 81, 74, and 45 percent at 10 years [11]. Many of the aforementioned studies have examined prostate biopsies following primary cryotherapy. Negative biopsy rates range from 87% to 93% [13, 15, 24].

In a recently published report, Donnelly et al. compared primary cryotherapy with external beam radiation in a randomized prospective trial of 244 patients designed to show noninferiority (defined as a 10% difference in disease progression) [25]. Progression was based on a trifecta definition including biochemical failure (defined by both Phoenix criteria as well as 2 consecutive rises with final value >1.0), radiologic evidence for metastases, and initiation of additional prostate cancer therapy. Secondary endpoints were overall and disease-specific survival and prostate biopsy at 3 years. Patients had T2 or T3 disease with the majority being either at high or intermediate risk, and all 122 patients received neoadjuvant hormone androgen deprivation therapy as per standard EBRT protocol, then progressing to treatment in each arm. At a median of 84-month followup, biochemical failure rates were similar with 27% in the cryotherapy arm versus 31.7% in the EBRT arm (Phoenix criteria); however, noninferiority could not be established because the small sample size resulted in wide confidence intervals. There was no difference in overall or disease-specific survival at 5 years, and there were significantly less positive biopsies at 3 years for cryotherapy (7.7% versus 28.9%). This must be interpreted cautiously as the clinical importance of positive biopsy after EBRT is unknown [26]. It should also be noted that the trial protocol was modified for the dose of neoadjuvant hormones from 3 months to 6 months, as well as radiation dose from 68 to 73.5 Gy, reflecting changing standards of practice. This may have an effect on biochemical failure rates on longer followup. 

## 6. Complications

When cryotherapy of the prostate was originally introduced in the 1960s, its use was limited and eventually abandoned due to the high morbidity of the procedure, particularly the high incidence of rectourethral fistula. With its reintroduction in the 1990s, three generations of improvements, and the various technological advancements, the adverse side effects associated with the procedure significantly decreased. 

The most dreaded complication, rectourethral fistula, is no longer expected with 0%–0.5% incidence during primary therapy, a rate similar to that seen for rectal injury following radical prostatectomy or radiation therapy [15, 16, 24, 27]. The use of thermocouple technology to monitor temperatures at the external sphincter has minimized rates of urinary incontinence to 1%–8%, previously as high as 83% with earlier systems [18, 28–31]. The updated COLD registry data presented in 2010 by Dhar et al. reported 3.1% incontinence at 12-months followup [32]. Urethral sloughing is minimized with the urethral warming catheter, and most series report rates from 1 to 15 percent, with resolution after catheterization and TURP necessary in the minority of cases [21].

Erectile dysfunction remains the major morbidity of cryotherapy, and it continues to be common despite technological improvements. Despite some series reporting rates of only 47%–50% [25], most series range between 75% to 93%, with the caveat that various definitions for erectile dysfunction with and without validated instruments were used [15, 16, 21]. Even recent series using third generation systems only report rates of 87% and 88% [29, 31]. One report by Ellis found that the use of a penile rehabilitation program improved their potency rates from 41% at 1 year to 51% at 5 years [18]. Nevertheless, given the high rates of erectile dysfunction, most reserve cryotherapy for those patients who are not concerned with potency.

Transient adverse effects such as urinary retention and rectal pain were minimal in most current series, occurring in less than 5% [29, 30]. 

## 7. Focal Cryotherapy

Although the side effect profile of whole-gland cryotherapy of the prostate has improved, quality of life issues remain, particularly the high rates of erectile dysfunction. This is the result of the proximity of the neurovascular bundles to the prostatic capsule combined with the necessity of the ice ball to extend outside of the capsule. The incidence of unilateral or unifocal small-volume disease and possibility for subtotal treatment with preservation of the contralateral neurovascular bundle build the conceptual basis for focal cryotherapy. Preliminary results from series of low-risk patients show improvement in postoperative erectile function (65%–89%) with acceptable bDFS rates using varying definitions (80%–95%) [33–36]. However, long-term data are lacking, and appropriate patient selection and particularly the ability to identify a truly unilateral or unifocal cancer using current diagnostic modalities remain to be determined and are currently under investigation.

## 8. HIFU

High intensity focused ultrasound (HIFU) is a truly noninvasive treatment modality in that it requires no incision or puncture to access the prostate. Instead, high-energy ultrasound waves produced by a transrectal probe are absorbed by prostatic tissue converting them to thermal energy which raises the temperature upwards of 100 degrees Celsius and results in coagulative necrosis [37]. Damage is also mediated by cavitation energy resulting from the interaction between the ultrasound waves and microbubbles in the sonicated tissue, which also leads to coagulation necrosis [38]. The ultrasound can be precisely focused on a tissue target, sparing intervening tissue including the rectal wall. Moreover, unlike EBRT, HIFU may be repeated if there is a proven local recurrence. For this reason, there is much interest in this technology as a minimally invasive method to ablate prostate lesions with potentially minimal side effects.

Currently, there are two HIFU devices on the market, the Ablatherm (Edap-Technomed, Lyon, France) and the Sonablate (Focus Surgery, Indianapolis, IN, USA). Both devices have undergone several generations of development to include safety features such as rectal wall cooling and precise monitoring of rectal temperature. A few differences exist in the technologies: while the Ablatherm relies on a preset treatment algorithm which assumes a constant tissue threshold for absorption of lethal energy in all patients, the Sonablate's system allows for modification of energy exposure to the prostate during the procedure based on grey-scale changes seen on ultrasound. Both devices are widely used in Europe and Japan, but are not yet available in the US outside of clinical trials, which are ongoing.

The first reported use of HIFU in the prostate was by Madersbacher for benign prostatic hypertrophy in 1994 [39], followed by its use by Gelet et al. for prostate cancer as reported in 1996 [40]. Since then, a number of series from Europe and Japan have reported short-intermediate term results. 

## 9. Selection Criteria

Due to focal length of the probe, there is an upper limit to gland size of 40–50 cc, and there should be no calcifications >1 cm as these will obscure ultrasonographic visualization [41, 42]. In some cases transrectal resection of larger calcifications may be performed prior to treatment. As with cryotherapy, most investigators select patients with localized disease and who either preferred avoiding conventional treatment or whose comorbidities excluded major surgery or EBRT [43]. Mearinin et al. found the best results in patients with low to moderate risk, with high rates of treatment failure in the high-risk group [44].

## 10. Definition of Treatment Success

As in the case of cryotherapy, the HIFU literature is plagued by a lack of consensus on the definition of biochemical failure, and therefore, treatment success. Originally, Gelet et al. used posttreatment PSA < 4.0 with negative biopsy as a measure for complete response to treatment [40]. This has evolved from the use of more strict PSA cut-offs, ranging from 0.2 to 0.5, to most later reports adopting the ASTRO or Phoenix criteria much like the cryotherapy literature. However, some argue that because HIFU is an ablative treatment, it should be held to the stringent standards of radical prostatectomy (PSA < 0.2), while others suggest EBRT as the prostate remains in situ after HIFU [45, 46]. In an effort to standardize reporting of outcomes, an international consortium of HIFU investigators meeting in Stuttgart, Germany analyzed the various definitions to establish their association with future clinical failure, defined as any record of positive biopsy after HIFU, initiation of secondary prostate cancer treatment, radiographic evidence of metastases, or prostate cancer death [47]. They found the “nadir + 1.2” definition to be optimal for predicting failure, defining this as the “Stuttgart definition”. 

As expected and as seen with cryotherapy, nadir PSA has been shown to be a predictor of subsequent PSA stability and negative biopsy. Uchida et al. used ASTRO criteria to show that a nadir PSA of ≤0.2 resulted in 100% bDFS at 3 years [42]. This was recently confirmed by Ganzer et al. using the newly reported Stuttgart definition, with a PSA ≤ 0.2 resulting in 84% bDFS at median followup of 6 years [48].

## 11. Outcomes

The largest series is reported by Uchida et al. with 517 men, using the Sonablate device [46]. Patients with stage T1-T3 disease were treated using various generations of the device, and 66% of the men received neoadjuvant hormonal therapy for reasons not specified. bDFS was defined using Phoenix criteria in this series. At a mean followup of 2 years, bDFS rates according to D'Amico low-, intermediate-, and high-risk groupings were 84%, 64%, and 45%, respectively. 483 patients underwent posttreatment biopsy, and negative biopsy rate was 83%. At the 2010 AUA meeting, Uchida reported updated results from his series and demonstrated an improvement in his outcomes using the more recent technology, with bDFS rates in low-, intermediate-, and high-risk groups of 93%, 72%, and 58%, compared with 75%, 54%, and 43% in older models [49]. The longest followup is reported by Blana et al. at a mean of 6.4 years, using prototype and first-generation Ablatherm devices [50]. They treated 140 patients with low-intermediate risk disease (T1-2 disease, PSA < 15, and Gleason score ≤ 7), defining treatment failure using the Phoenix criteria, initiation of salvage therapy, or positive posttreatment biopsy. 16.4% of patients received neoadjuvant hormones to decrease prostate volume, and this was discontinued after treatment. Actuarial disease-free survival at 7 years was 59%, with bDFS of 69%. The median PSA nadir was 0.16, and negative biopsy rate was 86.4% in 132 patients. 

In 2006, Illing et al. proposed a standardized protocol for treating patients using the Sonablate's ability to manually modify treatment based on real-time changes seen on ultrasound imaging [51]. The group reported on 34 men and found a significantly lower mean PSA nadir, 0.15 versus 1.51, when the visually directed protocol was used as opposed to an algorithm-based approach.

## 12. Complications

As with cryotherapy, technological advancements have decreased morbidity seen with primary HIFU treatment, including thermocouplers, higher-frequency transducers, preservation of a 5-mm apical margin to prevent stress urinary incontinence, and rectal cooling devices [52].

The most dreaded complication is rectouretheral fistula, but with rectal cooling, this is rarely seen. Thuroff et al. reported on results from the multicenter Ablatherm trial [53], with 5 fistulas seen in 402 patients (1.2%). With rectal cooling, this decreased to 0.5%. In Uchida's series, there were 6 fistulas (0.9%), all of which were in patients who had greater than 2 HIFU sessions [46]. Blana's series reported 0 fistulas, as did several other investigators [50, 54–56]. 

Post-HIFU stricture is a bothersome complication that may require serial dilations or TUIBNC. Uchida reported 16.6% stricture rate in his series, with 3.3% requiring TURP [46]; other authors record rates of 3.6% to 13.6% [50, 53, 54]. Chaussy reported a significant decrease in stricture and urine retention rate by TURP just before HIFU treatment with the Ablatherm device [57]. 

De novo erectile dysfunction is also known to occur, but as in the cryotherapy literature, rates are difficult to compare between series due to variable definitions and lack of use of validated instruments as well as differential use of neo-adjuvant hormonal therapy. Nevertheless, rates ranging from 20% to 43% have been reported [46, 50, 52, 56].

Low-grade incontinence was seen at higher rates in earlier series, with Thuroff et al. reporting 10.6% with Grade I and 2.5% Grade II [53], but this has decreased with next-generation HIFU devices, with grade I incontinence at 0.8% [46]. Prolonged urinary retention can occur after HIFU secondary to edema or urethral sloughing, and is reported to be 0.3 to 13.2% of cases and is usually resolved after recatheterization. Sloughing occurs in 9%–14% and is usually self-limited or managed using catheterization but may rarely require transurethral debridement. Infectious complications have also been reported at low rates including UTI and epididymitis [49, 53–56]. 

## 13. Vascular-Targeted Photodynamic Therapy

Vascular-targeted photodynamic therapy (VTP) is an investigational ablative technology which employs the use of a photosensitizing agent, which is taken up by tissue and produces radical oxygen species upon exposure to light which results in the destruction of the tissue [58]. Photosensitizers currently under investigation are WT-09 and WST-11, which remain confined to the vascular bed [59, 60]. Under ultrasound guidance, light is delivered via optical fibers inserted into the prostate using a standard brachytherapy grid, selectively effecting vascular occlusion and resultant death of prostatic tumors. Thus far, there are published results from a multi-center collaboration from Canada examining the safety and efficacy of VTP using WST-09 in the salvage setting after failed EBRT [61]. They published results from phase I/II studies which have shown a 60% complete response as seen on MRI as well as negative biopsy at 6 months in those subjects receiving the maximal dose of light energy, with no significant change in urinary or erectile function at 6 months. There are no published studies of VTP in the primary setting, but a prospective, multicenter phase I/II trial is currently underway.

## 14. Conclusion

Although the standard of care for prostate cancer continues to be surgical extirpation or EBRT, the downward stage migration resulting from aggressive screening has lead to a shift in priorities for many patients, with increasing interest in minimizing the side effects associated with traditional definitive treatment. Minimally invasive therapies such as cryotherapy and HIFU show promise, but continue to be plagued by a lack of long-term followup and consistency in outcomes reporting. With maturation of the cryotherapy and HIFU data sets, treatment-specific goals of therapy and a better understanding of side effect profiles may become available, and as investigational therapies such as VTP are further elucidated, clinicians and patients will hopefully be able to rely on these minimally invasive ablative therapies as a definitive treatment for prostate cancer.
